# PLK4 inhibitor exhibits antitumor effect and synergizes sorafenib via arresting cell cycle and inactivating Wnt/β-catenin pathway in anaplastic thyroid cancer

**DOI:** 10.1080/15384047.2023.2223383

**Published:** 2023-06-23

**Authors:** Wei Zhu, Bin Xie

**Affiliations:** Department of Pathology, Xiangya Hospital, Central South University, Changsha, China

**Keywords:** anaplastic thyroid cancer, polo-like kinase 4 inhibitor, sorafenib, malignant behaviors, synergistic effect

## Abstract

The anti-tumor effect of polo-like kinase 4 (PLK4) inhibitor has been explored in several solid carcinomas, while its application in anaplastic thyroid cancer (ATC) remains scarce. Hence, the current study aimed to investigate the effect of PLK4 inhibitor on the malignant behaviors of ATC cell lines and its synergistic antitumor effect with sorafenib. C643 and 8305c cells were cultured in various concentrations of centrinone (PLK4 inhibitor) with or without sorafenib. Meanwhile, the cell viability, cell apoptosis, cell cycle and expressions of glycogen synthetase kinase beta (GSK3β), p-GSK3β, β-catenin were determined. PLK4 mRNA and protein expressions were higher in most ATC cell lines than the normal thyroid epithelial cell line (all *P* < .05). Centrinone decreased cell viability, induced cell apoptosis, arrested cell cycle at G2/M phase and inactivated Wnt/β-catenin signaling with dose-dependent manners in C643 and 8305c cells (all *P* < .05). Interestingly, centrinone plus sorafenib further improved antitumor effect (*P* < .05 at most concentrations), with the highest combination index at 5 nM centrinone plus 4 μM sorafenib in C643 cells, then 4 nM centrinone plus 4 μM sorafenib in C643 cells. Subsequently, centrinone plus sorafenib reduced cell viability, promoted cell apoptosis, facilitated cell cycle at G2/M phase and repressed Wnt/β-catenin signaling more effectively compared with centrinone or sorafenib monotherapy in C643 and 8305c cells (all *P* < .05). PLK4 inhibitor exhibits antitumor effect and synergizes sorafenib via arresting cell cycle and inactivating Wnt/β-catenin pathway in ATC.

## Introduction

Anaplastic thyroid cancer (ATC), accounting for only 1% to 2% of thyroid cancer, is a highly aggressive, undifferentiated thyroid malignancy.^1,2^ Meanwhile, it is worth noticing that ATC is responsible for nearly 50% of the thyroid cancer-related death in 2020.^3^ Currently, the management for ATC includes surgical resection, hyper-fractionated accelerated external beam radiotherapy, targeted inhibitors, chemoradiation and palliative treatment, etc.^2,4^ Worse still, even applying the appropriate management, the prognosis of ATC remains poor with a 1-year overall survival rate ranging from 15% to 20%^5^. Subsequently, it is crucial to explore novel drugs and new treatment strategy to improve the prognosis of ATC patients.

Polo-like kinase 4 (PLK4), a family member of polo-like kinases (PLKs), consists of an N-terminal kinase domain and a C-terminal Polo-box domain (PBD).^6^ Preceding studies illuminate that the PLK4 modulates the centriole duplication and therefore plays a crucial role in tumor growth, invasion, and metastasis in a series of solid carcinomas.^7,8^ In detail, PLK4 knockdown *in vitro* inhibits the cancer cell growth in breast cancer cell line^7^. Besides, another study illuminates that PLK4 inhibits the apoptosis of tumor cell by stimulating phosphatidylinositol 3-kinase/protein kinase B (PI3K/Akt) signaling pathway in neuroblastoma cell lines.^8^

More importantly, PLK4 inhibitor has been applied in a variety of solid carcinomas such as osteosarcoma, lung carcinoma and pancreatic carcinoma.^9–11^ For instance, PLK4 inhibitor suppresses the osteosarcoma cell proliferation and induces the cell apoptosis in osteosarcoma or pancreatic carcinoma.^10,11^ Additionally, PLK4 inhibitor suppresses the cell viability in lung carcinoma cells, and inhibits the tumor growth in murine model of lung carcinoma^9^. Aforementioned evidences indicate PLK4 inhibitor elicits its antitumor effect in several carcinomas, while its involvement in ATC remains elusive.

Hence, the current study aimed to explore the effect of PLK4 inhibitor on the malignant behaviors of ATC cell lines and its synergistic antitumor effect with sorafenib.

## Methods

### Cell culture

Human ATC cell line C643 was purchased from CLS Cell Lines Service GmbH (CLS, Germany). Human ATC cell lines including 8505c, 8305c and CAL−62 were bought from Leibniz Institute DSMZ-German Collection of Microorganisms and Cell Cultures GmbH (DSMZ, Germany). Human ATC cell line KHM−5 M was obtained from JCRB cell bank (JCRB, Japan). Human normal thyroid epithelial cells were purchased from Cell Biologics, Inc. (CellBiologics, USA). The C643, 8505c 8305c and KHM−5 M cells were cultured in RPMI 1640 medium (Hyclone, USA) supplemented with 10% fetal bovine serum (FBS, Hyclone, USA). The CAL−62 cells were cultured with 10% FBS-containing DMEM (Hyclone, USA). The human normal thyroid epithelial cells were cultured with complete human epithelial cell medium (CellBiologics, USA). The expression of PLK4 in cells was evaluated (human normal thyroid epithelial cells were served as control).

### PLK4 inhibitor treatment

The centrinone (PLK4 inhibitor) and volasertib (PLK1 inhibitor) were purchased from GLPBIO Technology LLC (GLPBIO, USA). The C643 or 8305c cells were cultivated with various concentrations of centrinone and volasertib for 72 h (h). The cell viability, cell apoptosis, cell cycle and expressions of glycogen synthetase kinase beta (GSK3β), p-GSK3β, β-catenin were evaluated. The concentrations of centrinone for C643 cells were 0, 2.5, 5, 10, 20, 40 and 80 nM. The concentrations of centrinone for 8305c cells were 0, 2, 4, 8, 16, 32 and 64 nM. The concentrations of volasertib for C643 and 8305c cells were 0, 2, 4, 8, 16, 32 and 64 nM. The determination of the drug concentration gradient of centrinone and volasertib in c643 and 8305c cell lines was based on our preliminary experiment.

### Sorafenib treatment

The sorafenib was purchased from GLPBIO Technology LLC (GLPBIO, USA). Firstly, the C643 or 8305c cells were incubated with series concentrations (0, 1, 2, 4, 8, 16 and 32 μM) of sorafenib for 72 h. Then, the cell viability assessment was conducted. Secondly, the sorafenib (0, 1, 4, 16 μM) mixing with centrinone was cultured with C643 or 8305c cells for 72 h. The concentrations of centrinone for C643 or 8305c cells were 0, 5, 20 nM or 0, 4, 16 nM. Then, the cell viability was assessed and combination index was evaluated. Thirdly, sorafenib alone, contrinone alone and sorafenib + centrinone were incubated with C643 or 8305c cells for 72 h. The concentration of sorafenib for C643 and 8305c cells was 4 μM. The concentrations of contrinone for C643 and 8305c cells were 5 nM and 4 nM, respectively. The cell viability, cell apoptosis, cell cycle and expressions of GSK3β, p-GSK3β, β-catenin were determined.

### Reverse transcription quantitative polymerase chain reaction (RT-qPCR)

Trizol (Beyotime, China) was used to isolate total RNA. Then, the total RNA was transcribed to cDNA by reverse transcription master kit (Takara, Japan). Afterwards, the qPCR was performed with the application of qPCR kit (Takara, Japan). The thermal cycling was as follows: 1 cycle, 95°C for 30 s (s); 40 cycles, 95°C for 5 s and 61°C for 10 s. The primers (5’-> 3’) were as follows: PLK4, forward GCCTTATCACCTCCTCCTTCTG, reverse TGTAGTTGTAAGACCAAGTCCTTCA; GAPDH, forward GAGTCCACTGGCGTCTTCAC, reverse ATCTTG AGGCTGTTGTCATACTTCT. The data were calculated by 2^−ΔΔCt^ method.

### Western blot

The cells were lysed by RIPA buffer (Beyotime, China). The total protein was quantified by BCA kit (Sangon, China). The total protein with amount of 20 μg was separated by 4–20% precast gel (Beyotime, China) and transferred to nitrocellulose membrane (Sigma, USA). The membrane was blocked by 5% BSA (Beyotime, China) at 37°C for 1 h, incubated with diluted primary antibody at 4°C overnight, incubated with secondary antibody at 37°C for 1 h, successively. At last, the protein bands were developed by ECL kit (Sangon, China) and quantified by Image J 1.8.0 (NIH, USA). The primary antibodies were listed as follows: PLK4 antibody (1:1000, Invitrogen, USA), GSK3β antibody (1:3000, Invitrogen, USA), p-GSK3β (1:1000, Invitrogen, USA), β-catenin (1:1000, Invitrogen, USA), GAPDH antibody (1:5000, Invitrogen, USA). The secondary antibody was HPR linked goat anti-rabbit IgG antibody (1:200000, Invitrogen, USA).

### Cell viability, cell apoptosis and cell cycle

The cell viability was detected by cell counting kit−8 (Sangon, China). About 10 μl CCK−8 reagent mixing with 100 μl RPMI 1640 medium was added and incubated with cells for 2 h at 37°C. Then a microplate reader (BioTek, USA) was adopted to read optical density value. The cell apoptosis determination was conducted by TUNEL Apoptosis Assay Kit (Beyotime, China). In brief, the cells were fixed with 4% paraformaldehyde (Beyotime, China) for 30 min at room temperature. Then the cells were incubated with working solution at 37°C for 1 h. An inverted fluorescence microscope (Motic, China) was applied to capture images. Cell Cycle Analysis Kit (Beyotime, China) was applied to complete cell cycle analysis and the kit’s protocol was strictly followed. A flow cytometer (BD, USA) was used to determine the cells, and data were analyzed by Flowjo 7.6 (BD, USA).

### Statistical analysis

The data analysis and graph plotting were conducted by GraphPad Prism 7.01 (GraphPad Software, USA). The data were exhibited as mean and standard deviation. The comparisons among groups were evaluated by one-way ANOVA followed by Tukey’s or Dunnett’s multiple comparisons test. *P* value > 0.05, <0.05, <0.01, and < 0.001 were respectively marked as NS, *, **, and ***.

## Results

### PLK4 expression in ATC cell lines

**Figure 1.**
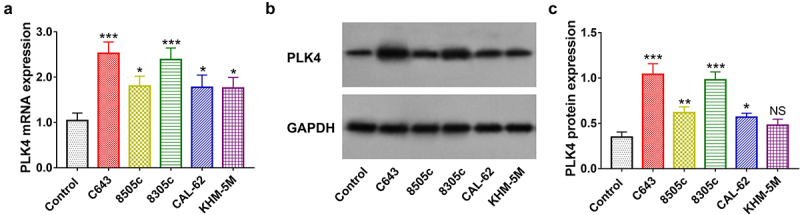
PLK4 expression in ATC cell lines. PLK4 mRNA expression. (a) Representative images of PLK4 protein expression by western blot assay. (b) and PLK4 protein expression. (c) in ATC cell lines. *: *P* < .05; **: *P* < .01; ***: *P* < .001, NS: no significance.

PLK4 mRNA and protein expressions were elevated in most ATC cell lines compared with control cell line (all *P* < .05, Figure 1a-c). In addition, the C643 and 8305c cells were chosen for subsequent study since they exhibited highest PLK4 mRNA and protein expression among all tested ATC cell lines.

### The effect of centrinone on ATC cell viability, cell apoptosis, cell cycle and Wnt/β-catenin signal pathway

**Figure 2.**
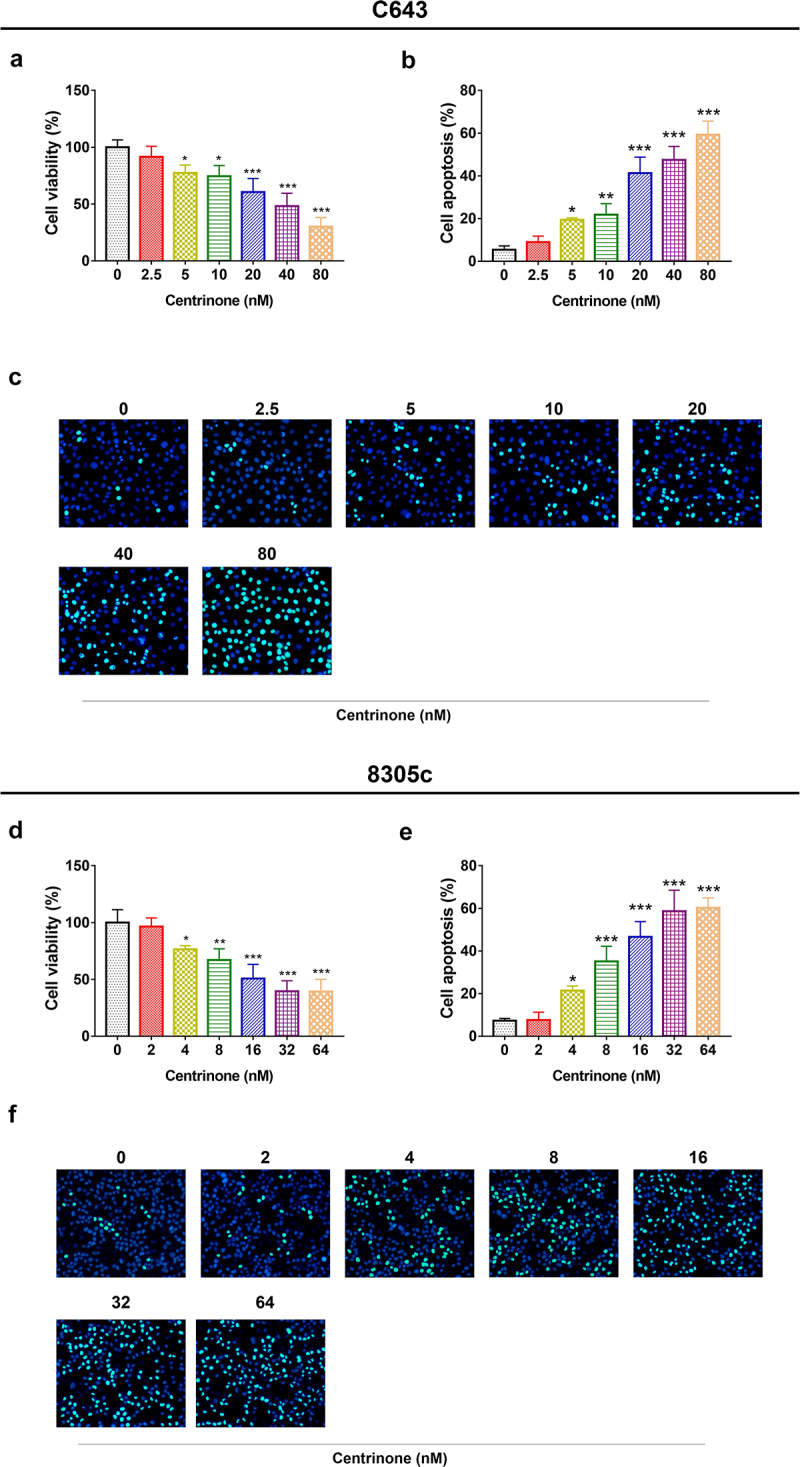
Cell viability and apoptosis in C643 and 8305c cells after treatment with different doses of centrinone. The C643 cell viability. (a) and apoptosis (b–c) after different doses of centrinone; the 8305c cell viability. (d) and apoptosis. (e–f) under different doses of centrinone. *: *P* < .05; **: *P* < .01; ***: *P* < .001.

**Figure 3.**
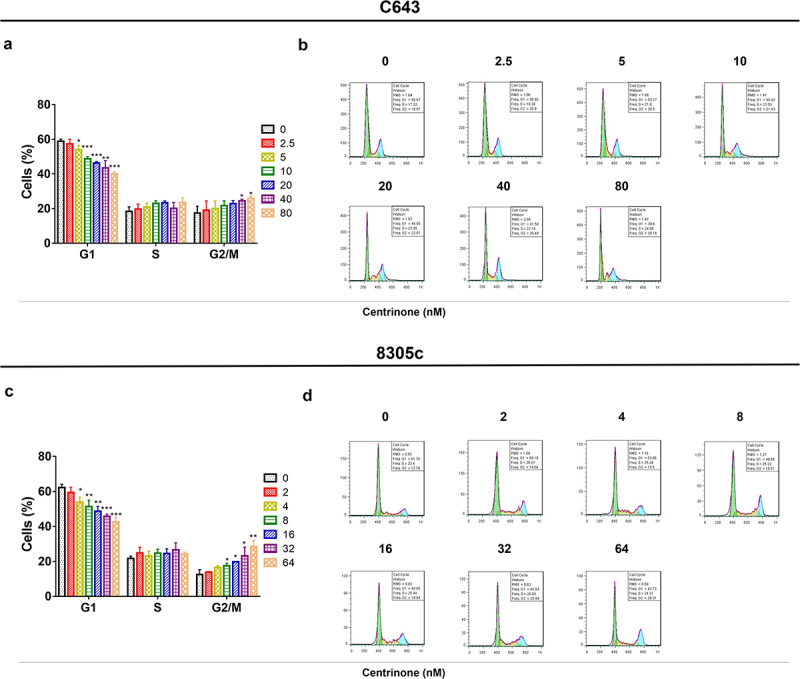
C643 and 8305c cells arrested at G2/M phase after treatment with centrinone. The percentage of C643. (a–b) and 8305c. (c–d) cells in G1, S and G2/M phase under different dose of centrinone. *: *P* < .05; **: *P* < .01; ***: *P* < .001.

In C643 cells: centrinone exhibited an effect on repressing the cell viability, enhancing the cell apoptosis, blocking the cell cycle at G2/M phase, inducing the phosphorylation of GSK3β and inhibiting β-catenin expression in a dose-dependent manner (all *P* < .05); besides, these effects of centrinone on 8305c cells showed the similar trends (all *P* < .05) (Figures 2a–f, figure 3 a–d, 4a–d). These data indicated that centrinone disclosed an anti-tumor effect via arresting the cell cycle at G2/M phase and downregulating Wnt/β-catenin signaling in ATC cell lines.

**Figure 4.**
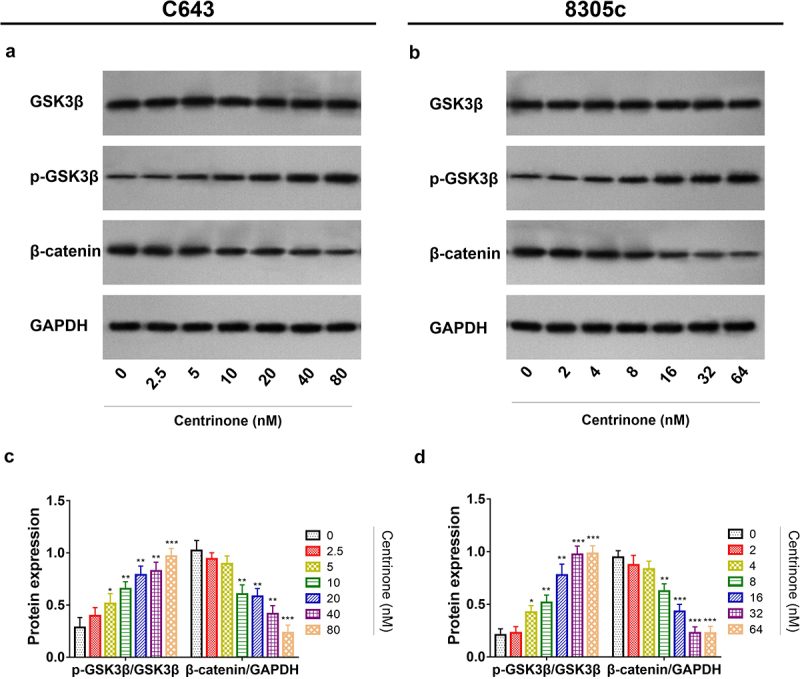
Centrinone inhibited the activation of Wnt/β-catenin signaling. Representative images of GSK3β, pGsk3β, β-catenin expressions under different doses of centrinone by western blot assay in C643 cell line. (a) and in 8305c cell line. (b). Expressions of pGsk3β/GSK3β and β-catenin/GAPDH under different doses of centrinone in C643 cell line (C) and in 8305c cell line. (d). *: *P* < .05; **: *P* < .01; ***: *P* < .001.

### The synergistic effect of centrinone and sorafenib

**Figure 5.**
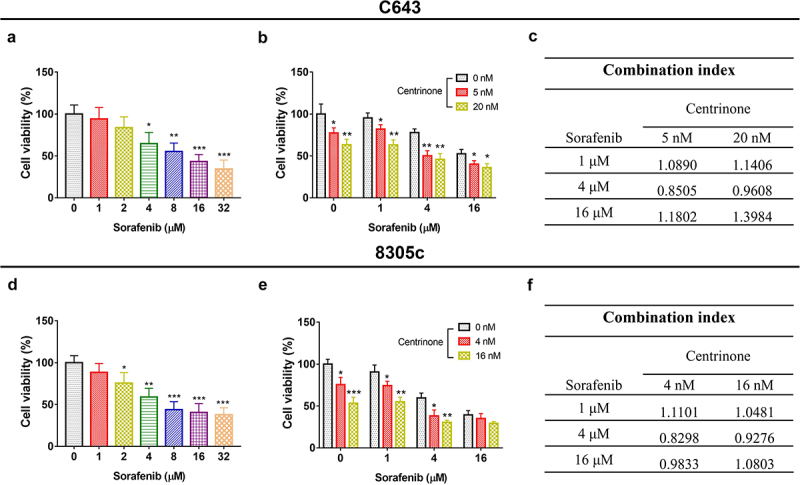
The synergistic effect of centrinone and sorafenib. Cell viability of C643 cells affected by sorafenib at different dose (a); Cell viability of C643 cells affected by the combination of centrinone and sorafenib (b); Combination index (CI) of the centrinone plus sorafenib in C643 cell line (c); Cell viability of 8305c affected by sorafenib (d); Cell viability of 8305c affected by the combination of centrinone and sorafenib (E); CI of the centrinone plus sorafenib in 8305c cell line (f). *: *P* < .05; **: *P* < .01; ***: *P* < .001.

In C643 cells, the cell viability decreased under sorafenib treatment in a dose-dependent manner (all *P* < .05, Figure 5a). Besides, the combination of centrinone and sorafenib under different concentrations further reduced cell viability compared to monotherapy (all *P* < .05, Figure 5b). Subsequently, 5 nM centrinone and 4 μM sorafenib showed the maximum synergy with a combination index of 0.8505, thereby chosen for following study (Figure 5c).

In 8305c cells, the cell viability declined under sorafenib treatment in a dose-dependent manner (all *P* < .05, Figure 5d). Besides, the combination of centrinone plus sorafenib under different concentrations further downregulated cell viability compared to monotherapy (all *P* < .05, Figure 5e). Correspondingly, 4 nM centrinone and 4 μM sorafenib disclosed the maximum synergy with a combination index of 0.8298 and were selected for subsequent study (Figure 5f). Furthermore, other potential synergy pathways were also discovered on the Search Tool for Interacting Chemicals database (STITCH) (available at: http://stitch.embl.de/)^12^ and the Kyoto Encyclopedia of Genes and Genomes (KEGG) database (available at: https://www.kegg.jp/). Then, it was shown that sorafenib might regulate the FoxO signaling pathway (**Supplementary Table S1**). Besides, the PLK4 was also involved in this pathway (**Supplementary Figure S1**). It was hypothesized that PLK4 inhibitor and sorafenib might exhibit the synergy effect through the FoxO signaling pathway. However, this finding needed further exploration.

### The effect of centrinone plus sorafenib on ATC cell viability, cell apoptosis, cell cycle and Wnt/β-catenin signal pathway

**Figure 6.**
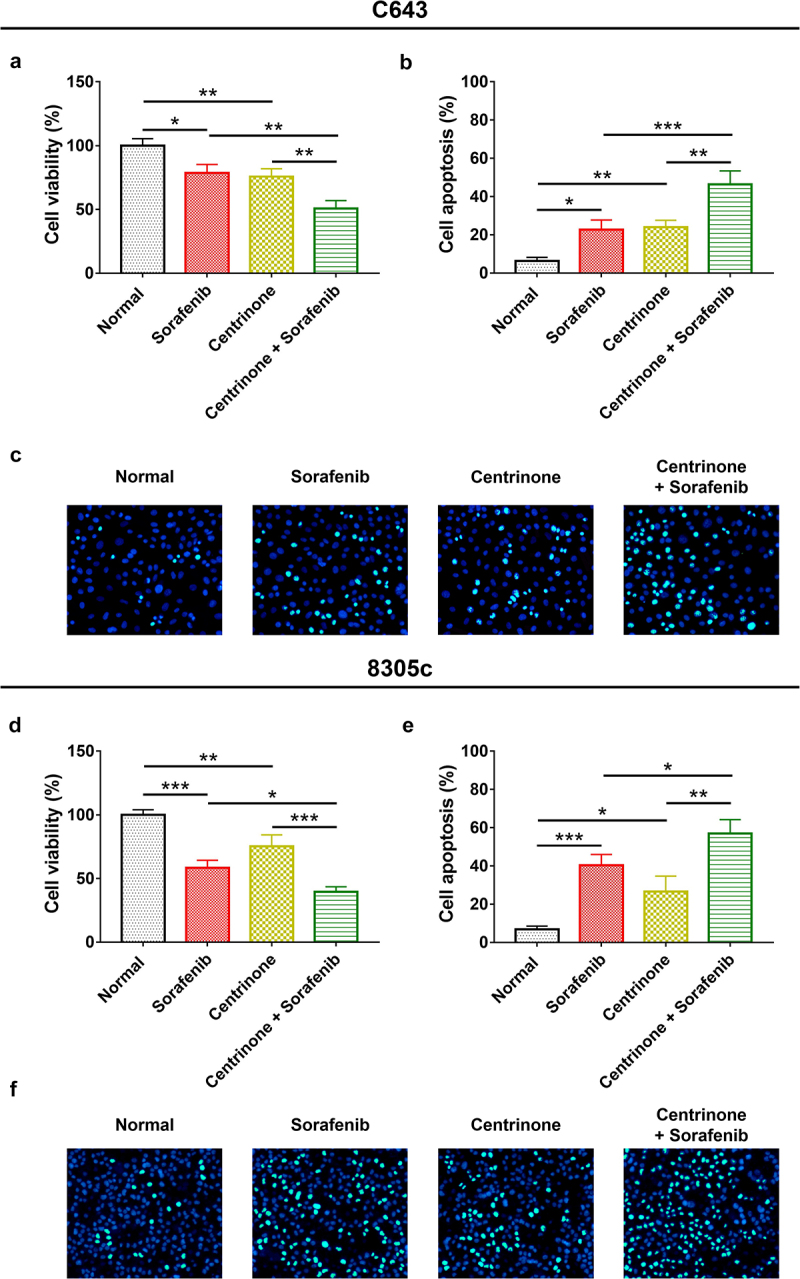
Cell viability and apoptosis in C643 and 8305c cell lines after the treatment with centrinone and sorafenib. The cell viability (a) and apoptosis (B-C) of C643 cells treated by centrinone and sorafenib; the cell viability (d) and apoptosis (e–f) of 8305c treated by centrinone and sorafenib. *: *P* < .05; **: *P* < .01; ***: *P* < .001.

**Figure 7.**
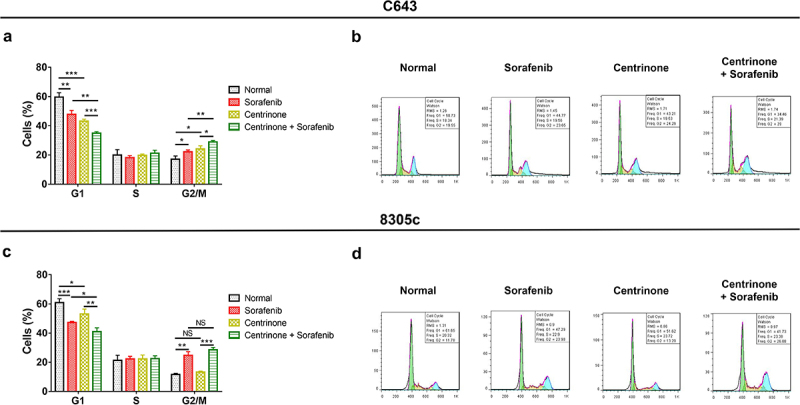
C643 and 8305c cells arrested at G2/M phase after centrinone and sorafenib. The percentage of C643 (A-B) and 8305c (C-D) cells in G1, S and G2/M phase treated by centrinone and sorafenib. *: *P* < .05; **: *P* < .01; ***: *P* < .001, NS: no significance.

**Figure 8.**
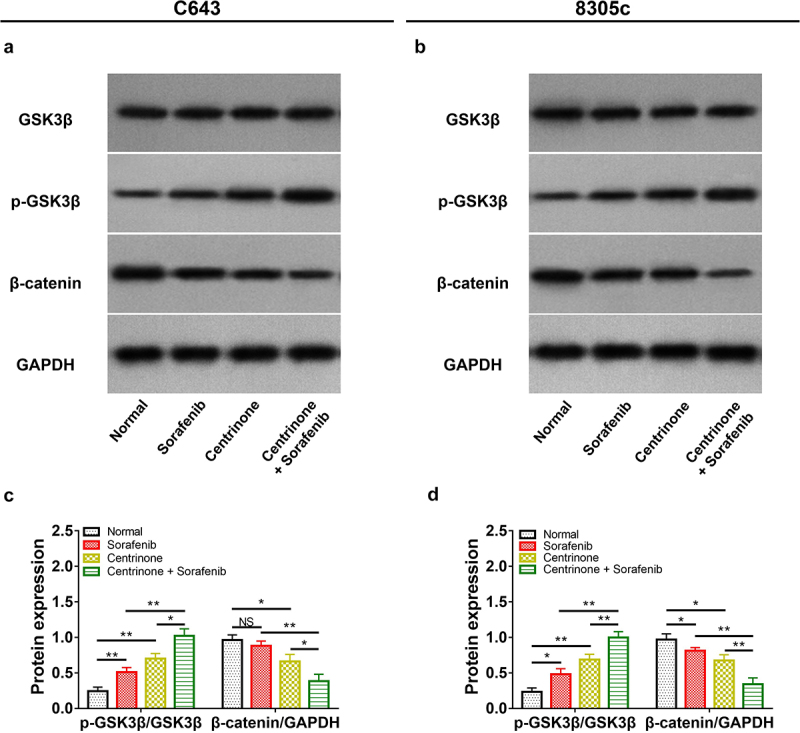
Centrinone and sorafenib inactivated Wnt/β-catenin signaling. Representative images of GSK3β, pGsk3β, β-catenin expressions by western blot assay in C643 cell line (A) and in 8305c cell line (B) treated by centrinone and sorafenib. Expressions of pGsk3β/GSK3β and β-catenin/GAPDH in C643 cell line (C) and in 8305c cell line (D) treated by centrinone and sorafenib. *: *P* < .05; **: *P* < .01.

In C643 cells, centrinone plus sorafenib showed a better effect on decreasing cell viability, promoting cell apoptosis, blocking the cell cycle at G2/M phase, downregulating Wnt/β-catenin signaling compared with centrinone alone or sorafenib alone (all *P* < .05). Besides, the similar trend was also observed in 8305c cells (all *P* < .05), except that G2/M cell proportion was of no difference between centrinone plus sorafenib regimen and sorafenib regimen alone (Figures 6a–f, 7 a–d, 8a–d).

### Comparison of the effect of centrinone with volasertib on ATC cells

It was reported that the signal pathways targeted by the PLK1 inhibitor and PLK4 inhibitor were similar; so, it may be meaningful to compare which was more effective in ATC therapy. To clarify this issue, the efficacy of volasertib in ATC cells was discovered, which indicated that the volasertib could also inhibit cell viability and promote cell apoptosis in a dose-dependent manner in treating the c643 cells (**Supplementary Figure S2a–c**). Furthermore, similar trends were also observed in the 8305c cells (**Supplementary Figure S2d–f**). Besides, the IC_50_ was similar between the centrinone and volasertib in ATC cells (**Supplementary Figure S3**). Then, it might be preliminarily concluded that the efficacy was similar between PLK1 inhibitor and PLK4 inhibitor in ATC cells.

## Discussion

Currently, a previous study has elucidated that volasertib (PLK1 inhibitor) decreases the cell viability of 8505c, 8305c, and KAT18 cell lines (ATC cell lines) in a dose-dependent manner.^13^ While in terms of PLK4 inhibitor, its apoptotic effect is explored in merely a few other solid carcinomas but not ATC^7,14,15^. For instance, one previous study elucidates that CFI−400945 (a PLK4 inhibitor) suppresses the proliferation and facilitates the apoptosis of rhabdoid tumor cell lines.^14^ Another illustrates that CFI−400945 suppresses breast cancer cell proliferation *in vitro* and inhibits tumor growth *in vivo*.^15^ What’s more, it is also illustrated that YLT−11 (PLK4 inhibitor) suppresses breast cancer growth in xenograft models.^7^ In the current study, PLK4 inhibitor exhibited a capability in decreasing ATC cell viability, while promoting cell apoptosis. Possible explanation could be that 1) PLK4 inhibitor might inhibiting the activation of several cancer-related signaling pathways (such as Wnt/β-catenin pathway), which further suppress ATC cell viability.^16^ 2) PLK4 inhibitor arrests cell cycle at G2/M phase in ATC cell lines as illustrated in our study, thus leading to a suppression of ATC cell viability.

Sorafenib is a widely used multi-kinase inhibitor that targets both the RAF/MEK/ERK pathway and receptor tyrosine kinases, thereby further inhibits tumor growth and angiogenesis in several tumors^17^. Recently, sorafenib has been approved in treatment of ATC, whose combination with several antitumor therapies exhibits a synergistic effect in treating ATC.^13,18,19^ For instance, a previous study illustrates that sorafenib plus N-Hydroxy−7-(2-Naphthylthio) and heptanomide exhibits a synergistic effect in suppressing ATC cell viability and increasing its apoptosis.^19^ Besides, another study discloses that sorafenib synergizing with paclitaxel and radiation therapy suppresses cell viability and induces cell apoptosis of 8505c cell line (ATC cell line).^18^ Similarly, it is also illuminated that sorafenib combined with volasertib (PLK1 inhibitor) represses cell viability via blocking cell cycle at G2/M phase, which further induces cell apoptosis of 8505c, 8305c, and KAT18 cell lines (ATC cell lines).^13^ Whereas, its synergistic effect with PLK4 inhibitor remains elusive. Correspondingly, in the current study, the combination of PLK4 inhibitor with sorafenib exhibited a better effect on decreasing cell viability, promoting cell apoptosis, arresting cell cycle at G2/M phase compared with PLK4 inhibitor alone or sorafenib alone in C643 and 8505c cell lines. Possible explanation could be that the synergistic antitumor effect of PLK4 inhibitor and sorafenib could be realized when they inhibit non-overlapping oncogenic pathways in ATC. Additionally, we indicate that combination of PLK4 inhibitor with sorafenib might reduce the dose of administration in ATC than that of either drug alone, which might further reduce the side effects.

Generally, the Wnt/β-catenin signal pathway is a well-known oncogenic pathway in ATC cell lines.^20–23^ To be specific, the phosphorylation of GSK3β disassociates β-catenin from the destruction complex in the cytosol, subsequently, the free β-catenin relocates in the nucleus and thus modulates the malignant behaviors of tumor cells.^24,25^ More importantly, it is revealed that Wnt/β-catenin signaling induces the activation of epithelial – mesenchymal transition (EMT) in ATC cell lines, which play important roles in migration and invasion of ATC cells.^26^ Besides, the inhibition of PLK4 suppresses tumor growth via deactivating Wnt/β-catenin signaling in colorectal cancer cell line.^27^ Whereas the effect of PLK4 inhibitor on Wnt/β-catenin signaling in ATC cell lines remains elusive. In the present study, it was disclosed that PLK4 inhibitor suppressed the activation of Wnt/β-catenin pathway in ATC cell lines, which is in accordance with some previous studies on other carcinomas.^27–29^ Besides, we hypothesize that the combination of PLK4 inhibitor and sorafenib might exhibit a synergistic effect on downregulating Wnt/β-catenin signaling compared with monotherapy. However, these study findings should be further verified in the *in vivo* study to elevate the possibility of the clinical application of this study’s discoveries.

Collectively, PLK4 inhibitor exhibits antitumor effect and synergizes sorafenib via arresting cell cycle and inactivating Wnt/β-catenin pathway in ATC, which provides a potential option for ATC treatment.

## Supplementary Material

Supplemental Material

## Data Availability

The original contributions presented in the study are included in the article, further inquiries can be directed to the corresponding author.
