# Supplementary material for: PLK4 inhibitor exhibits antitumor effect and synergizes sorafenib via arresting cell cycle and inactivating Wnt/β-catenin pathway in anaplastic thyroid cancer
Source: Cancer Biol Ther. 2023 Jun 23;24(1):2223383. doi: 10.1080/15384047.2023.2223383 (PMC10292002; doi:10.1080/15384047.2023.2223383)
Supplement: Supplemental Material [file KCBT_A_2223383_SM1557.zip › Supplementary material/Supplementary table 1.docx]

**Supplementary table 1.** KEGG pathway analysis for sorafenib.

| #pathway ID | pathway description | observed gene count | false discovery rate |
| --- | --- | --- | --- |
| 4015 | Rap1 signaling pathway | 18 | 2.34E-26 |
| 4014 | Ras signaling pathway | 17 | 5.66E-24 |
| 5200 | Pathways in cancer | 17 | 2.73E-21 |
| 4151 | PI3K-Akt signaling pathway | 17 | 4.75E-21 |
| 4510 | Focal adhesion | 15 | 7.34E-21 |
| 5205 | Proteoglycans in cancer | 11 | 5.25E-13 |
| 4060 | Cytokine-cytokine receptor interaction | 11 | 3.14E-12 |
| 5206 | MicroRNAs in cancer | 9 | 1.96E-11 |
| 4370 | VEGF signaling pathway | 7 | 9.13E-11 |
| 5214 | Glioma | 7 | 1.05E-10 |
| 5211 | Renal cell carcinoma | 7 | 1.35E-10 |
| 4540 | Gap junction | 7 | 8.13E-10 |
| 5215 | Prostate cancer | 7 | 8.90E-10 |
| 5221 | Acute myeloid leukemia | 6 | 4.09E-09 |
| 4144 | Endocytosis | 8 | 6.53E-09 |
| 4810 | Regulation of actin cytoskeleton | 8 | 1.16E-08 |
| 4917 | Prolactin signaling pathway | 6 | 1.30E-08 |
| 5218 | Melanoma | 6 | 1.30E-08 |
| 5161 | Hepatitis B | 7 | 1.99E-08 |
| 4012 | ErbB signaling pathway | 6 | 3.62E-08 |
| 4915 | Estrogen signaling pathway | 6 | 6.88E-08 |
| 4062 | Chemokine signaling pathway | 7 | 9.65E-08 |
| 4722 | Neurotrophin signaling pathway | 6 | 2.17E-07 |
| 4919 | Thyroid hormone signaling pathway | 6 | 2.17E-07 |
| 5220 | Chronic myeloid leukemia | 5 | 6.86E-07 |
| 4010 | MAPK signaling pathway | 7 | 8.69E-07 |
| 5219 | Bladder cancer | 4 | 2.92E-06 |
| 4916 | Melanogenesis | 5 | 3.24E-06 |
| 4660 | T cell receptor signaling pathway | 5 | 3.47E-06 |
| 4066 | HIF-1 signaling pathway | 5 | 4.30E-06 |
| 4650 | Natural killer cell mediated cytotoxicity | 5 | 1.01E-05 |
| 5223 | Non-small cell lung cancer | 4 | 1.16E-05 |
| 4910 | Insulin signaling pathway | 5 | 1.34E-05 |
| 5212 | Pancreatic cancer | 4 | 1.79E-05 |
| 4720 | Long-term potentiation | 4 | 1.98E-05 |
| 4664 | Fc epsilon RI signaling pathway | 4 | 2.32E-05 |
| 5203 | Viral carcinogenesis | 5 | 5.61E-05 |
| 4912 | GnRH signaling pathway | 4 | 6.62E-05 |
| 5216 | Thyroid cancer | 3 | 6.62E-05 |
| 4670 | Leukocyte transendothelial migration | 4 | 0.000178 |
| 4068 | FoxO signaling pathway | 4 | 0.000219 |
| 5213 | Endometrial cancer | 3 | 0.000402 |
| 4921 | Oxytocin signaling pathway | 4 | 0.000526 |
| 4730 | Long-term depression | 3 | 0.000599 |
| 5120 | Epithelial cell signaling in Helicobacter pylori infection | 3 | 0.000824 |
| 4662 | B cell receptor signaling pathway | 3 | 0.000963 |
| 5100 | Bacterial invasion of epithelial cells | 3 | 0.00109 |
| 5169 | Epstein-Barr virus infection | 4 | 0.00109 |
| 4914 | Progesterone-mediated oocyte maturation | 3 | 0.00135 |
| 4640 | Hematopoietic cell lineage | 3 | 0.00164 |
| 4666 | Fc gamma R-mediated phagocytosis | 3 | 0.00172 |
| 4114 | Oocyte meiosis | 3 | 0.00288 |
| 5166 | HTLV-I infection | 4 | 0.00288 |
| 4726 | Serotonergic synapse | 3 | 0.00315 |
| 5160 | Hepatitis C | 3 | 0.00494 |
| 5034 | Alcoholism | 3 | 0.0059 |
| 4630 | Jak-STAT signaling pathway | 3 | 0.00811 |
| 4020 | Calcium signaling pathway | 3 | 0.0113 |
| 5210 | Colorectal cancer | 2 | 0.0164 |
| 4920 | Adipocytokine signaling pathway | 2 | 0.0221 |
| 4520 | Adherens junction | 2 | 0.0237 |
| 5323 | Rheumatoid arthritis | 2 | 0.034 |
| 4750 | Inflammatory mediator regulation of TRP channels | 2 | 0.0406 |
