# Supplementary figures and images for: PLK4 inhibitor exhibits antitumor effect and synergizes sorafenib via arresting cell cycle and inactivating Wnt/β-catenin pathway in anaplastic thyroid cancer
Source: Cancer Biol Ther. 2023 Jun 23;24(1):2223383. doi: 10.1080/15384047.2023.2223383 (PMC10292002; doi:10.1080/15384047.2023.2223383)

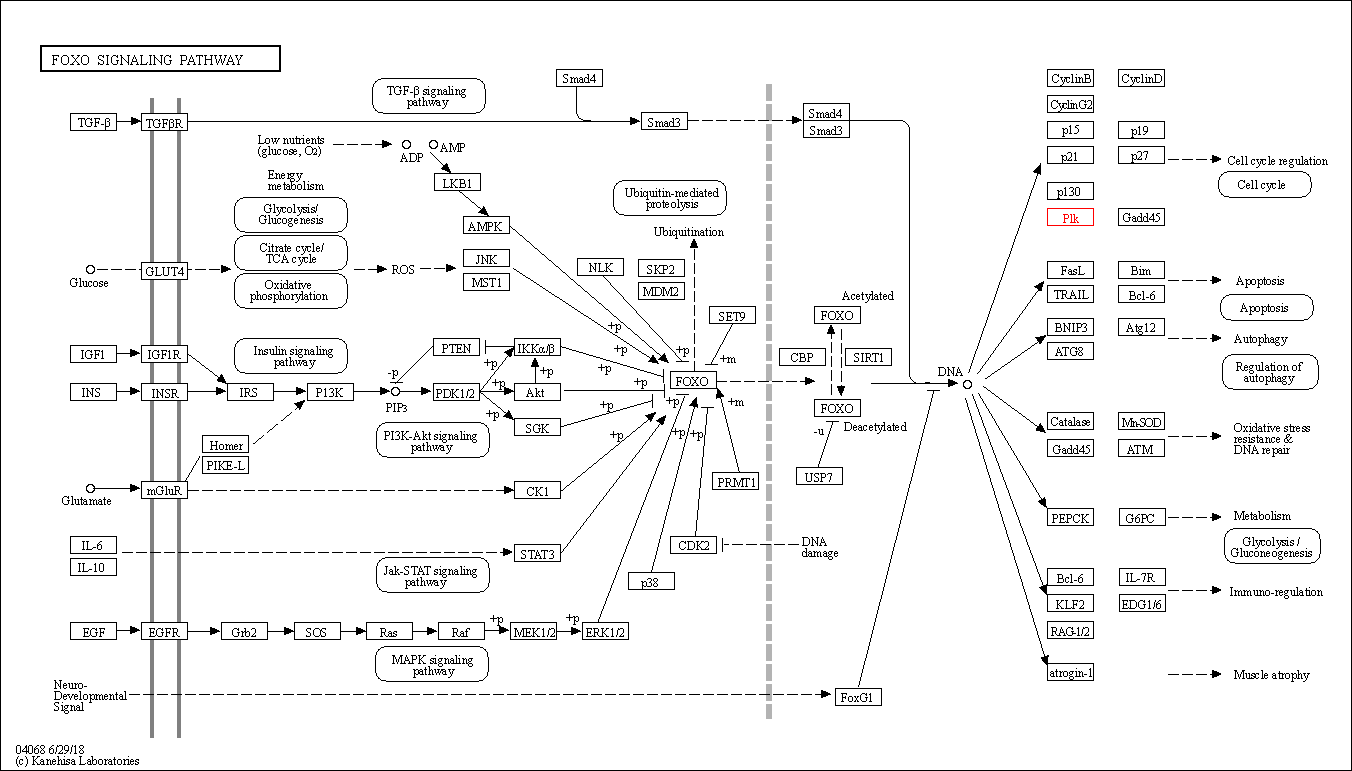

Supplement: Supplemental Material [file KCBT_A_2223383_SM1557.zip › Supplementary material/Supplementary figure 1.tif]

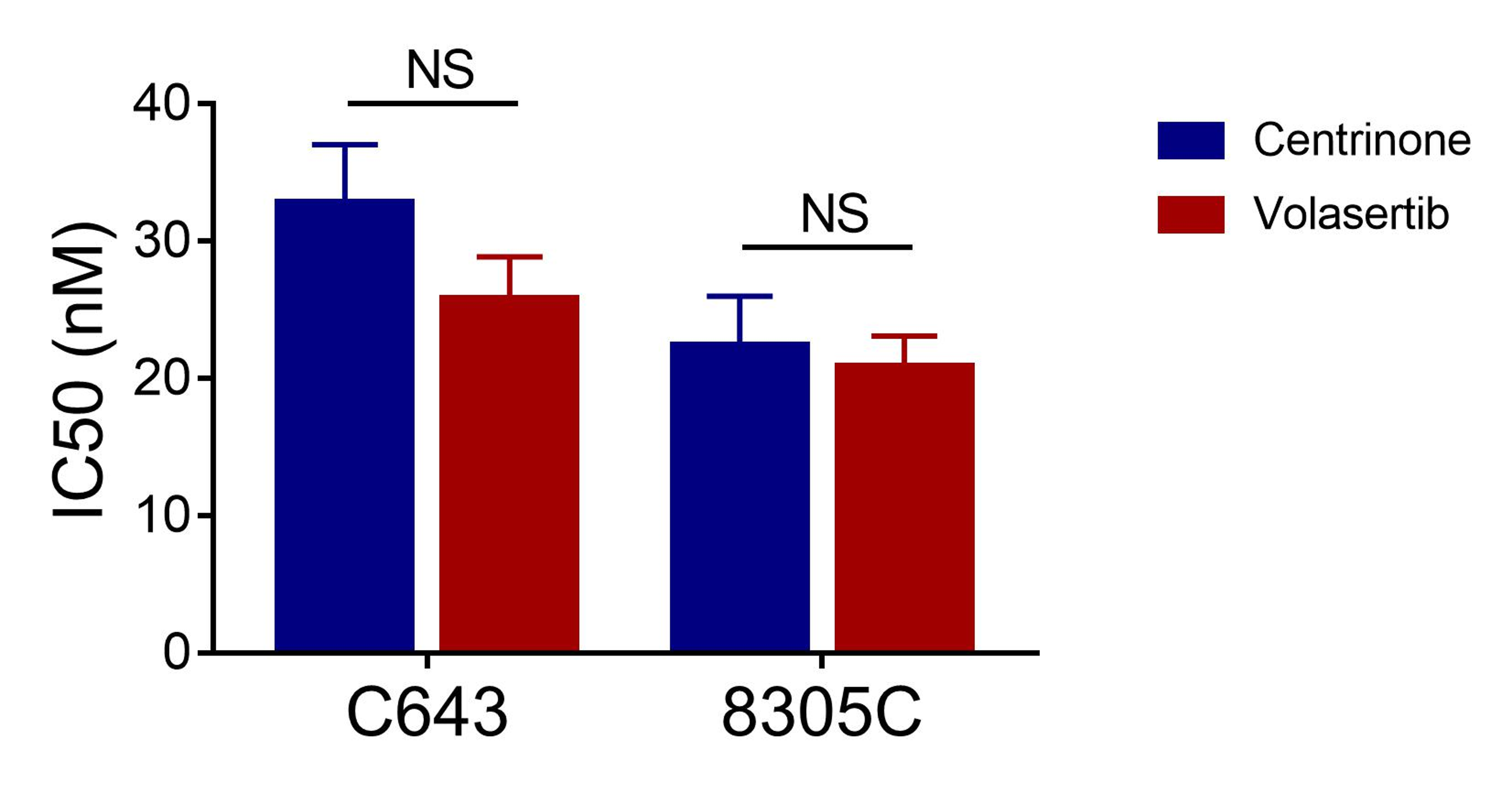

Supplement: Supplemental Material [file KCBT_A_2223383_SM1557.zip › Supplementary material/Supplementary figure 3.tif]
